# Association of KIF6 Variant with Lipid Level and Angiographic Coronary Artery Disease Events Risk in the Han Chinese Population

**DOI:** 10.3390/molecules170911269

**Published:** 2012-09-21

**Authors:** Ge Wu, Gui-Bin Li, Bin Dai

**Affiliations:** The Forth Hospital of Jilin University, Changchun 130011, Jilin, China; Email: wugerde@163.com (G.W.); 857582559@sina.com (B.D.)

**Keywords:** KIF6, rs20455, cardiovascular risk, plasma lipoprotein level, Han populations

## Abstract

KIF6 is a class of molecular motor from the kinesin superfamily. Recently, multiple large studies consisting mainly of Europeans have shown that KIF6 Trp719Arg SNP may be a new predictive factor for coronary artery disease (CAD) event risk. The allelic frequency distribution of rs20455 is different in various populations, yet studies among the Han population, one of the largest ethnic groups in the World, have not been conducted*.* This study is aimed to evaluate the association of KIF6 Trp719Arg variant with angiographic CAD and serum lipid levels in the Han population from northern China. In this case-controlled study, peripheral blood samples were collected from 356 patients and 568 controls of Han Chinese origin. Genotyping was performed by a high-resolution melting curve. The impact of rs20455 on CAD and non-fatal MI was evaluated in a dominant genetic model with stepwise multiple regression analysis. There were no significant differences of genotypes and allele frequency between angiographic CAD and control groups (*p* > 0.05); however, that of MI and non-MI subgroups were significant differences (*p* < 0.05). After adjusting for significant risk factors, angiographic CAD risk was not significantly increased in 719Arg allele carriers compared with non-carriers. Further analysis revealed that the non-fatal MI risk and triglyceride levels were significantly higher in 719Arg allele carriers than non-carriers. In conclusion, KIF6 719Arg allele was not an independent risk factor for angiographic CAD susceptibility in Han populations from northern China. However, it was associated with a significantly higher TG level, which may indicate an increased myocardial infarction risk in angiographic CAD patients.

## 1. Introduction

Coronary artery disease (CAD) is influenced by both environmental and genetic factors [1,2]. In China, morbidity and mortality associated with CAD is increasing, and is affecting younger populations [3]. Recently, many studies have examined genetic polymorphisms associated with CAD [4]. There are several CAD susceptibility gene loci that have been verified by Genome Wide Association Studies (GWAS); however, many of these results are based on European Caucasians. Among the identified mutations, only chromosome 9p21 is associated with CAD risk in the Chinese Han population, with the carriers having a 30% increased risk of CAD compared with non-carriers [5]. Kinesin-Like Protein 6 (KIF6) is a new candidate gene for CAD which was first identified in 2007 as a potential risk factor in European populations [6]. It is a class of homodimeric molecules involved in intracellular microtubule transportation and is ubiquitously expressed in coronary arteries and other vascular tissue [7]. The expression level of KIF6 has been reported to be higher in healthy homozygous carriers of the chromosome 9p21 CAD risk allele than in non-carriers of the 9p21 risk allele [8]. However, no studies have yet reported a link between KIF6 and CHD events in the Chinese Han population. 

From 2008 to 2010, there were multiple large prospective and case-control studies on the association of KIF6 Trp719Arg polymorphism with CAD risk. These studies included the cholesterol and recurrent events trial (CARE), including 3,847 Caucasian patients; the West of Scotland coronary prevention study (WOSCOPS) trials [9], a case-control study of over 1,700 Caucasian participants; the Atherosclerosis Risk in Communities (ARIC) study [10], a large prospective study of 15,000 Americans; the Women’s Health Study (WHS) [11], a large prospective study of over 25,000 healthy white females; the Cardiovascular Health Study (CHS) [12], a prospective study in older North Americans; and the Pravastatin or Atorvastatin Evaluation and Infection Therapy—Thrombolysis In Myocardial Infarction 22 (PROVE IT–TIMI22) study [13]. All of these trials reported that the KIF6 Trp719Arg polymorphism is significantly, and independently, associated with increased coronary events risk and statin beneﬁt. Similarly, a meta-analysis of seven prospective studies [14] demonstrated that carriers of the KIF6 719Arg allele (Arg/Arg homozygotes and Arg/Trp heterozygotes), but not non-carriers (Trp/Trp homozygotes), were at an increased risk of CAD and received a significant benefit from statin therapy. 

However, in recent studies, consisting primarily of Europeans, conflicting results have been reported regarding the association between rs20455 and CAD. Data from a meta-analysis involving 17,000 CAD cases and 39,000 controls from 19 different studies powerfully refuted the rs20455 link to CAD [15]. Also, findings from the Wellcome Trust Case Control Consortium (WTCCC) [16,17] showed no association of the rs20455 polymorphism with CAD. A case-control study of a Costa Rican population [6] showed that there was no link between KIF6 Trp719Arg polymorphism and non-fatal myocardial infarction (MI). Similarly, in the Heart Protection Study (HPS) [18] and the Intervention Trial Evaluating Rosuvastatin (JUPITER) trial [19], even the correlation between KIF6 Trp719Arg variant and statin response had became debatable.

These contradictory conclusions demand evaluation of the predictive ability of this new marker of cardiovascular disease in underrepresented ethnic groups. Importantly, the genotype distribution and allelic frequency of KIF6 Trp719Arg polymorphism has been shown to vary between ethnic groups. The Chinese Han population is one of the World’s largest races, but there have not been related studies on this population. In this case-control study, for the first time, we aim to investigate the KIF6 Trp719Arg polymorphism distribution, its effect on lipid metabolism and association of KIF6 719Arg allele with risk of coronary events (CAD/non-fatal MI) in the Han Nationality from northern China.

## 2. Results and Discussion

### 2.1. Baseline Clinical Characteristic 

Relevant baseline clinical characteristics and traditional risk factors of the participants are shown in Table 1. In all variables, significant differences were observed in age (*p* < 0.01), smoking status (*p* < 0.05), diabetes (*p* < 0.01), HDL-cholesterol (*p* < 0.05), MI (*p* < 0.001), angina (*p* < 0.001) in angiographic CAD cases *versus* controls.

**Table 1:** General characteristics of angiographic CAD cases and controls.

General Characteristics	Control (n = 568)	Cases (n = 356)	
***Age***	***60.3 ± 12.1***	***64.9 ± 11.31 ^b^***	
Female (%)	55.6	47.2	
*Non-alcohol (%)*	*81.2*		*70.6*
*Active alcohol (%)*	*17.6*		*28.1*
Former alcohol (%)	1.2		1.3
***Non-smokers (%)***	***63***		***47.9 ^a^***
***Active smokers (%)***	***33.1***		***48.3 ^a^***
Former smokers (%)	3.9		3.8
***Diabetes (%)***	***11.27***		***26.9 ^b^***
Hypertension (%)	61.8		69.7
TC (m mol/L)	5.29 ± 1.04		5.41 ± 1.15
Triglycerides (m mol/L)	2.15 ± 1.66		2.16 ± 1.77
***HDL-cholesterol (m mol/L)***	***1.36 ± 0.32***	***1.26 ± 0.31 ^a^***	
LDL-cholesterol (m mol/L)	3.35 ± 0.75	3.27 ± 0.75	

^a^ *p* < 0.05; ^b^ *p* < 0.01; ^c^ *p* < 0.001; HDL, high density lipoprotein; LDL, low density lipoprotein.

### 2.2. Frequency Distribution of Genotypes and Alleles in Angiographic CAD/Non-fatal MI Patients and Controls

**Figure 1:**
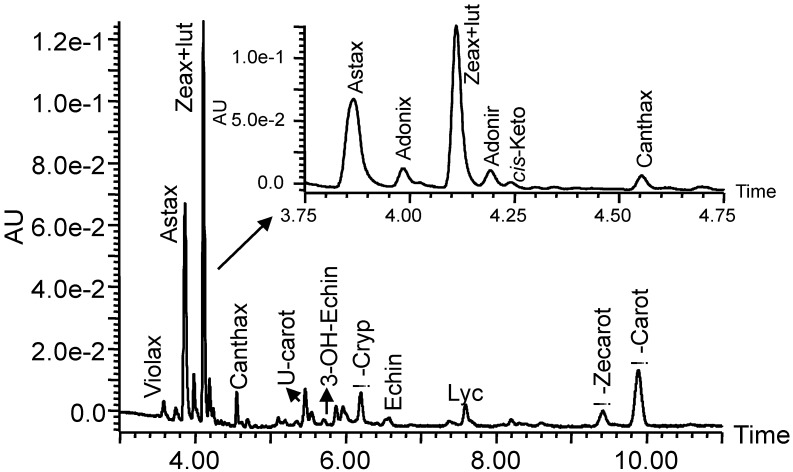
A typical picture of melting curves and genotype graphs of rs20455 by HRM. (**A**) melting curve; (**B**) genotype graph; green curve—Trp/Trp; red curve—Arg/Arg; blue curve—Trp/Arg.

924 participants were successfully genotyped for the KIF6 Trp719Arg polymorphism (Figure 1). As shown in Table 2, the genotype distribution of KIF6 Trp719Arg polymorphism was in Hardy-Weinberg equilibrium (*p* = 0.982). Among controls, the genotype frequencies were Trp/Trp 29.6%, Arg/Trp 47.2% and Arg/Arg 23.2%; whereas in angiographic CAD patients, the genotype frequencies were 29.2%, 46.1% and 24.7%, respectively. There were no statistically significant differences in genotype frequencies between angiographic CAD cases and the control group (*p* > 0.05). The frequency of 719Arg was not significantly different between angiographic CAD patients (47.8%) and controls (46.8%) (OR = 1.0158, 95% [CI] = 0.7797–1.3234, *p* = 0.9203).

The angiographic CAD patients were further divided into MI and non-MI subgroups. As shown in Table 2, the genotype frequencies within the MI subgroup were Trp/Trp 14.0%, Arg/Trp 59.7% and Arg/Arg 26.3%; whereas in the non-MI subgroup, frequencies were 36.4%, 39.7%, and 24.0%, respectively. There was a statistically significant difference in genotype frequencies between these two subgroups (*p* < 0.05). The frequency of 719Arg was significantly different in MI patients (56.1%) compared with non-MI patients (43.8%) (OR = 1.5942, 95% [CI] = 1.0167–2.4992, *p* = 0.041). Also, there were significant differences in the minor allele frequency between MI (56.1%) and Non-MI + control subgroups (45.9%) (OR = 1.5073, 95% [CI] = 1.0154–2.2369, *p* = 0.0409).

### 2.3. Identification of Independent Traditional Risk Factors Associated with Angiographic CAD Risk

To identify traditional risk factors for angiographic CAD, stepwise multiple regression analysis was performed, which included the variables shown in Table 3. This analysis revealed that age (OR = 1.035, 95% [CI] = 1.011–1.059, *p* = 0.004), smoking status (OR = 1.889, 95% [CI] = 1.098–3.253, *p* = 0.021) and diabetes (OR = 2.908, 95%[CI] = 1.444–5.854, *p* = 0.002) were major independent risk factors for angiographic CAD in these populations, while the plasma HDL-cholesterol levels were a protective factor (OR = 0.276, 95% [CI] = 0.094–0.814, *p* = 0.020).

### 2.4. Association of KIF6 Trp719Arg with Angiographic CAD Risk Adjusted for Established Risk Factors

As shown in Table 2, after adjusting for established risk factors in our populations, binary logistic regression analysis revealed that the 719Arg carriers were not at significantly increased risk of angiographic CAD (adjusted OR = 1.0177, 95% [CI] = 0.7608–1.3614, *p* = 0.9203) compared with non-carriers.

### 2.5. Association of KIF6 Trp719Arg with Non-Fatal MI Risk in Angiographic CAD Subjects

As shown in Table 2, after adjusting for established risk factors in our populations, binary logistic regression analysis revealed a significant increase in risk for MI (adjusted log-dominant mode of inheritance = 3.1625, 95% [CI] = 1.3706–7.2969, *p* < 0.0001) in carriers of the KIF6 719Arg allele, compared with non-carriers between MI and non-MI subgroups. On comparison of the MI and non-MI + control, statistically significant differences in MI risk were also evident (adjusted log-dominant mode of inheritance = 2.6303, 95% [CI] = 1.4349–4.8002, *p* < 0.0001).

### 2.6. Association of KIF6 Trp719Arg with Plasma Lipid Level in Different Genotypes

As shown in Table 4, within the whole study population, TG levels in carriers of 719Arg variant were significantly greater when compared to non-carriers (*p* < 0.05). In controls, LDL-cholesterol levels in carriers of 719Arg variant were significantly higher when compared to non-carriers (*p* < 0.05). In contrast, no significant difference was observed in TC or HDL-cholesterol levels among genotypes in all groups (*p* > 0.05).

**Table 2:** Relative risk of events estimated with binary regression analyses.

genotype	n	Trp/Trp	Trp/Arg	Arg/Arg	MAF	719Trp *versus* 719Arg (allelic)		Adjusted log-dominant mode		
		n (%)	n (%)	n (%)		OR (95% CI)	*P* Value	OR (95% CI)	*p* Value	
CAD	356	104 (29.21)	164 (46.07)	88 (24.72)	0.4775	1.0158 (0.7797–1.3234)	0.9203	1.0177 (0.7608–1.3614)	0.9204	
**control**	568	168 (29.58)	268 (47.18)	132 (23.24)	0.4683	ref_1_		ref_1_		
**MI**	114	16 (14.03)	68 (59.65)	30 (26.32)	0.5614	1.5942 (1.0167–2.4992)	0.0410	3.1625 (1.3706–7.2969)	<0.0001	
						1.5073 (1.0154–2.2369)	0.0409	2.6303 (1.4349–4.8002)	<0.0001	
**Non-MI**	242	88 (36.36)	96 (39.67)	58 (23.97)	0.4380	ref_2_		ref_2_		
**Non-MI + control**	810	256 (31.60)	364 (44.94)	190 (23.46)	0.4593	ref_3_		ref_3_		

OR = odds ratio; 95% CI = 95% conﬁdence interval; CC = homozygous for the minor allele; CT = heterozygotes; MAF = minor allele frequency; TT = homozygous for the major allele. Allele frequencies were estimated by direct counting. Adjusted for age, gender, smoking status, diabetes and plasma HDL cholesterol. ref_1_ = CAD *versus* control. ref_2_ = MI *versus* non-MI (upper line). ref_3_ = MI *versus* non-MI + control.

**Table 3:** Odds Ratio of angiographic CAD estimated with univariate analyses.

Variables	*age*	female	Alcohol	*Smoking ever*	*Diabetes*	TC (m mol/L)	Triglycerides (m mol/L)	*HDL-cholesterol (m mol/L)*	LDL-cholesterol (m mol/L)
Odds Ratio **(95%CI)**	***1.035 (1.011–1.059)***	1.403 (0.824–2.388)	1.828 (0.971–3.441)	***1.889 (1.098–3.253)***	***2.908 (1.444–5.854)***	0.886 (0.688–1.140)	0.994 (0.852–1.160)	***0.276 (0.094–0.814)***	0.870 (0.607–1.246)
***p***	***0.004***	0.212	0.060	***0.021***	***0.002***	0.346	0.940	***0.020***	0.447

**Table 4:** Association of KIF6 Trp719Arg with plasma lipid level.

genotype	TC (m mol/L)		Triglycerides (m mol/L)		HDL-cholesterol (m mol/L)		LDL-cholesterol (m mol/L)	
	control	case	control	case	control	case	control	case
Trp/Trp	5.34 ± 1.20	5.57 ± 1.41	***2.43 ± 2.14 ^a^***	***2.51 ± 2.26 ^a^***	1.36 ± 0.32	1.27 ± 0.39	***3.28 ± 0.64 ^a^***	3.22 ± 0.82
Trp/Arg + Arg/Arg	5.20 ± 0.89	5.26 ± 0.83	***1.91 ± 1.08***	***1.86 ± 1.20***	1.37 ± 0.32	1.25 ± 0.22	***3.43 ± 0.84***	3.35 ± 0.74

^a^ *p* < 0.05 Trp/Trp *versus* Trp/Arg + Arg/Arg between angiographic CAD and controls.

### 2.7. Discussion

This is the first report on the association of KIF6 Trp719Arg polymorphism with angiographic CAD events in Han populations from northern China. Nine hundred and twenty four (924) participants were successfully genotyped for the KIF6 Trp719Arg polymorphism. To our knowledge, this is the first publication describing HRM analysis as a detection method for the rs20455 polymorphism. As a new detection method, HRM analysis is associated with several advantages over traditional genotype analysis, including faster speed, simple operation, high throughput, high sensitivity, high specificity, and limited pollution. In our case-control study, the minor allelic frequency of rs20455 is 0.478, which is lower than Sub-Saharan African populations (0.908) or Han Chinese (0.556), but it is higher than in European (0.358) and Japanese (0.386) populations [20,21]. The genotype distribution of rs20455 in northern Chinese participants deviated slightly from HWE expectations (*p* > 0.05). When evaluating the difference in genotype between control and angiographic CAD groups, we failed to find a significant difference in genotype distribution and allele frequencies of KIF6 Trp719Arg SNP. Binary regression analysis also revealed that 719Arg allele may not be an independent risk factor for angiographic CAD in the Han people of northern China. In a subgroup analysis restricted to the angiographic CAD group, on the effect of 719Arg allele on non-fatal MI risk, the genotype distribution and minor allele frequency showed significant differences. We also found that the susceptibility of non-fatal MI was increased significantly compared with non-carriers independent of various risk factors such as age, sex, and smoking status. 

Kinesin is a molecular motor that was discovered in squid and mammalian nervous tissue by Vale *et al.* in 1985 [20,22,23]. The KIF6 gene spans a genomic region of about 390,000 base pairs on human chromosome 6p21 containing 23 exons and 22 introns [24]. Trp719Arg polymorphism (rs20455) is a common polymorphism in the KIF6 gene that leads to an arginine (Arg) substitution for tryptophan (Trp) at position 719. After it was identified as a potential risk factor for CAD in Europeans, the Celera Corporation has heavily marketed its KIF6 Trp719Arg variant assay to cardiologists and primary care physicians [9,11,13,20,25]. Patient populations in studies that have investigated this association have consisted primarily of white Americans or Europeans, with only a small number of non-white patients represented. Nearly all studies demonstrated a modest increase in risk of CAD in carriers of the KIF6 719Arg variant allele (odds ratio [OR]: 1.1 to 1.5). The exact patho- physiologic role of the Trp719Arg polymorphism in CAD has been widely speculative. Some trials claim that Trp719Arg replaces a nonpolar residue with a basic residue near the putative cargo-binding domain and therefore might affect cargo binding [23]. Another study showed that the Trp719Arg polymorphism is associated with the presence of late outgrowth endothelial progenitor cells in acute MI [25], which may provide a link between KIF6 and the pathogenesis of CAD.

Results from several clinical studies have also contradicted the association between Trp719Arg and CAD [26]. In 2010, an elegant meta-analysis from 19 different studies (consisting primarily of European populations) strongly refuted the KIF6 association with angiographic CAD [15]. Data from the Ottawa Heart Genomic Study (OHGS) [27], a cross-sectional case-control study designed to examine the potential association between rs20455 SNP and MI, did not support the hypothesis that Caucasian carriers of the KIF6 719Arg allele have increased risk for angiographic CAD or MI. Further, among those without prior disease in PROSPER study [21], no significant benefit was observed in either carriers or noncarriers. Interestingly, the minor allele frequency and linkage equilibrium patterns for KIF6 differ markedly according to ancestry [11], which warrants research of this potential marker of cardiovascular disease in additional ethnic groups.

CAD is the second leading cause of cardiovascular death in China [28]. According to the latest survey from the WHO, the CAD subjects and death toll in China ranks second in the World. The prevalence of CAD induces a heavy financial burden to the nation and the prevention of CAD has aroused great concerns in Chinese society. In the HapMap database, the minor allele frequency of the Trp719Arg polymorphism in Chinese has been shown to be significantly different compared with Europeans (*p* > 0.05) [21]. To date there has been no study that has looked at the link between rs20455 and CAD events among Chinese.

Genetic polymorphisms associated with CAD events are primarily related to lipid metabolism of lipoproteins, apolipoproteins and enzymes involved in gene encoding. To assess whether an increased risk of MI/angina in carriers within the CAD population was associated with abnormal plasma lipid level, we performed analyses of the plasma lipid levels between genotypes. The recently published regression meta-analysis of 144,931 participants that included KIF6 negative studies (e.g., Assimes, HPS and JUPITER studies), reported that the 719Arg allele increased vulnerability to the harmful effect of LDL cholesterol on the risk of CVD [29]. Our results revealed that TG levels in carriers of the 719Arg allele were significantly higher than non-carriers, indicating that in Han populations from northern China, the rs20455 polymorphism may be involved in the regulation of lipid metabolism, which may enhance the MI risk in patients with CAD. However, further studies are warranted to elucidate the detailed physiological and pathological mechanisms associated with CAD. 

There were several limitations of this study. The small number of patients and unequal baseline data may have confounding effects. A larger sample size and comprehensive analysis of the interaction between genetic and environment factors may reveal additional insight into the role of rs20455 in the pathogenesis of CAD. Additionally, further study should be designed to test the association of KIF6 variants with statin response in Han Chinese.

## 3. Experimental

### 3.1. Study Population

The present case-control study included a cohort of 924 unrelated Han Chinese residents recruited from the Changchun, Jinlin area in Northern China. The cohort was comprised of 356 CAD subjects with the following inclusion criteria: coronary angiography of >50% stenosis in one or more arteries and stable or unstable angina. MI was defined by detection of elevated cardiac biomarkers (upper reference limit for creatine kinase-MB is 0–25 U/L or troponin is 0.1 ng/mL) together with evidence of myocardial ischemia [30]. Five hundred and sixty eight (568) controls were excluded from the CAD group by clinical symptoms and electrocardiography. After obtaining informed consent, all the individuals were personally interviewed for information including ethnicity, age, sex, smoking status and alcohol consumption. The study protocol was approved by the institutional review board of the Fourth Hospital of Jilin University in China.

### 3.2. Data Collection

Baseline clinical information was obtained by reviewing the subject’s medical records. Patients with diabetes mellitus were identified as having fasting plasma glucose >6.9 mmol/L, or using anti-diabetic medication. Hypertension was deﬁned as systolic blood pressure >140 mmHg, a diastolic blood pressure >90 mmHg, or patients with a documented diagnosis or using antihypertensive medication. All laboratory parameters were determined in fasting patients not using lipid-lowering medication for at least four weeks. All lipid levels were measured on Hitachi 7170 automatic biochemical analyzer. Total cholesterol, triglyceride were measured by enzyme method, reagent by DESAY diagnostic system (Shanghai, China) Limited company. LDL-C and HDL-C levels were detected by direct determination method, using reagents provided by Beijing Jiu Qiang Biological Limited (Beijing, China).

### 3.3. Genetic Analysis

Blood samples were drawn by vein-puncture after an overnight fast of 12 h. The samples were separated by centrifugation, and aliquots were stored at −86 °C until analyses. Genomic DNA was extracted following a standard protocol [31]. Primers were designed according to the U.S. National Center for Biotechnology Information (NCBI) primer-blast tool. The sense strand was 5'-CTGTGAAACTCCTTCTG-3' (17 bp), and the antisense strand was 5'-TGGCTTATCAAGAGACATGAGA-3' (22 bp). The PCR reaction consisted of the following components: 10 × PCR buffer 1 μL, MgCl_2_ (25 mM) 1 μL, dNTPs (2.5 mmol/L) 0.25 uL, Eva-green saturation dye (20×) 0.5 μL, primer (10 μmol/L) 0.25 μL, template DNA (10 ng/uL) 1 μL, Taq enzyme (5U) 0.1 μL, ultra pure water 5.65 μL. The expansion program was 95 °C for 5 min (pre-degenerated), 95 °C for 10 s, 60 °C for 15 s, 72 °C for 25 s (centigrade), for 50 cycles. Genotyping was performed by a high-resolution melting curve according to the Roche Light Cycler 480 protocol: 95 °C for 1 min, 40 °C for 1 min, 95 °C for detecting fluorescent 40 times per second, cool to 40 °C for 10 s.

### 3.4. Statistical Analysis

All data were analyzed using SPSS 17.0 (SPSS Inc, Chicago, IL, USA). Differences between baseline characteristics of participants were assessed by *t*-tests (for continuous variables), by chi-square statistics or Fisher’s exact test (for categorical variables), and reported as two-sided *p-*values. Two-sided *p*-values < 0.05 were considered statistically significant. Deviations from Hardy-Weinberg Equilibrium expectations were tested by the chi-square test. The gene counting method tested genotype distribution and allele frequencies [32]. The chi-square test was applied to evaluate differences in genotype distributions and allele frequencies between groups. Differences in plasma cholesterol concentrations among genotypes were analyzed with chi-square statistics. Stepwise multiple regression analyses were used to evaluate potential interactions between angiographic CAD events and traditional risk factors. Binary regression analysis was used to evaluate the impact of KIF6 gene polymorphism on angiographic CAD (angiographic CAD/non-fatal MI) adjusted for significant risk factors. The impact of KIF6 on outcomes was tested primarily in a dominant genetic model comparing carriers (Arg/Arg plus Trp/Arg) *versus* non-carriers (Trp/Trp).

## 4. Conclusions

In conclusion, our results supported the notion that carriers of the KIF6 719Arg allele are not associated with risk of angiographic CAD, but have an intrinsic correlation with an increased risk of non-fatal MI in angiographic CAD subjects of Han nationality from China. Carriers of the 719Arg allele have higher TG levels than non-carriers, indicating that KIF6 Trp719Arg SNP may be involved in lipid metabolism and may serve as a MI diagnostic biomarker in Northern Chinese populations. The functional research regarding the KIF6 variant with respect to angiographic CAD, and the benefit from statin therapy, awaits confirmation in future studies. Overall, these results may indicate that rs20455 could predict those at highest risk for MI, which may be important in pre-clinical diagnosis, early risk stratiﬁcation, early intervention, prognostication, or individualization of cardiovascular therapy. 
